# Indium gallium nitride-based ultraviolet, blue, and green light-emitting diodes functionalized with shallow periodic hole patterns

**DOI:** 10.1038/srep45726

**Published:** 2017-04-04

**Authors:** Hyun Jeong, Rafael Salas-Montiel, Gilles Lerondel, Mun Seok Jeong

**Affiliations:** 1 Laboratoire de Nanotechnologie et d’Instrumentation Optique, Institut Charles Delaunay, CNRS-UMR 6281, Université de Technologie de Troyes, BP 2060, 10010 Troyes, France; 2 Center for Integrated Nanostructure Physics (CINAP), Institute for Basic Science (IBS), Sungkyunkwan University, Suwon 440–746, Republic of Korea; 3 Department of Energy Science, Sungkyunkwan University, Suwon 440-746, Republic of Korea

## Abstract

In this study, we investigated the improvement in the light output power of indium gallium nitride (InGaN)-based ultraviolet (UV), blue, and green light-emitting diodes (LEDs) by fabricating shallow periodic hole patterns (PHPs) on the LED surface through laser interference lithography and inductively coupled plasma etching. Noticeably, different enhancements were observed in the light output powers of the UV, blue, and green LEDs with negligible changes in the electrical properties in the light output power versus current and current versus voltage curves. In addition, confocal scanning electroluminescence microscopy is employed to verify the correlation between the enhancement in the light output power of the LEDs with PHPs and carrier localization of InGaN/GaN multiple quantum wells. Light propagation through the PHPs on the UV, blue, and green LEDs is simulated using a three-dimensional finite-difference time-domain method to confirm the experimental results. Finally, we suggest optimal conditions of PHPs for improving the light output power of InGaN LEDs based on the experimental and theoretical results.

Gallium nitride (GaN)-based light-emitting diodes (LEDs) have recently become widespread in the fields of solid-state lighting, backlight units, automobile lighting, and outdoor full-colour displays because of their significantly lower energy consumption, longer lifetime, and higher brightness than conventional incandescent light bulbs.^1^,^2^,^3^,^4^,^5^,^6^,^7^,^8^ Moreover, the spectral range of indium gallium nitride (InGaN)/GaN multiple quantum wells (MQWs), which are typically used as active layers in InGaN-based LEDs, is from ultraviolet (UV) to green lights, with substantial light emission efficiency.^9^,^10^,^11^ However, the low-light extraction efficiency (LEE) of LEDs is still a significant issue to be solved.^12^,^13^,^14^ Therefore, assorted technologies, such as surface texturing and formation of micro/nanostructures, graded refractive index layers, embedded deflectors, self-assembled silica nanospheres, and photonic crystal slabs on the LED surface, were reported to overcome low LEE of InGaN-based LEDs.^15^,^16^,^17^,^18^,^19^,^20^,^21^,^22^ Among these proposed technologies, the use of periodic hole patterns (PHPs) on the LED is one of the most attractive methods because of its controllable, predictable, and large-scale process compatible characteristics. Moreover, the PHPs on the LED allow forming the uniform effective medium over the whole surface area. It has been reported that periodic patterns on GaN-based LED are more effective to enhance the light output power than non-periodic patterns such as a surface roughness due to light waveguide through the effective medium.^23^,^24^ To fabricate PHP on nanometer scale, nanoimprint lithography, direct holographic photodissolution, and laser interference lithography are commonly applied.^25^,^26^,^27^,^28^,^29^,^30^ According to previous reports on LEDs with PHPs, research groups mainly investigated the photonic crystal effect on LEE improvement.^31^,^32^,^33^,^34^. However, the depth of holes should be deeper than 100 nm to form a photonic band gap, which negatively influences the electrical properties of LEDs.^35^ Therefore, PHP with a shallow depth are required to avoid electrical loss in PHP-functionalized LEDs.

In this study, the effects of the emission wavelength and hole depth on the enhancement of the light output power of InGaN-based LEDs functionalized with PHPs were experimentally and theoretically investigated. InGaN-based UV, blue, and green LED epitaxial structures were grown on *c*-plane sapphire substrates through metal–organic vapour deposition. LED chips including a transparent current spreading layer and metal electrodes were prepared using conventional LED fabrication. Laser interference lithography and inductively coupled plasma (ICP) etching were conducted to obtain periodic holes on the LED surface. The morphology of UV, blue, and green LED surfaces was examined by implementing field-emission scanning electron microscopy (FE-SEM) and atomic force microscopy. The electrical properties and light output power of the LEDs with the PHPs were confirmed using a conventional probe station equipped with a photodiode. By using confocal scanning electroluminescence (EL) microscopy (CSEM), spatially resolved EL characteristics of the LEDs with the PHPs were studied. Moreover, three-dimensional (3D) finite-difference time-domain (FDTD) simulations were conducted to substantiate the experimental results.

## Results

**Figure 1:**
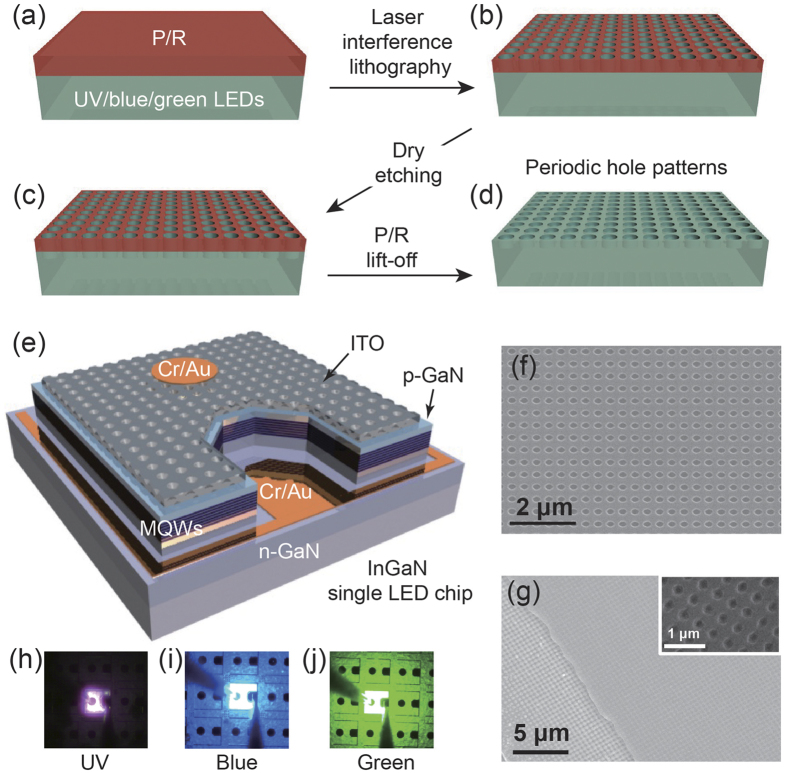
Fabrication of InGaN LEDs with PHPs. (**a**–**d**) 3D Schematics of the fabrication of a PHP on the LED surface by using laser interference lithography. (**e**) 3D illustration of a fully fabricated LED single chip with PHP. The plane-view SEM images of (**f**) the ITO transparent current-spreading layer placed on the top of the LEDs and (**g**) the edge region of ITO. The PHPs were formed on not only the ITO but also the p- and n-GaN layers. The inset shows the magnified SEM image of the PHP on ITO. The optical photographs for (**h**) the UV, (**i**) blue, and (**j**) green LEDs with the PHPs.

Figure 1(a–d) show the schematic of the PHP fabrication on the LED using laser interference lithography. First, a 500 nm thick PR solution was spread on the cleaned LED surface through typical spin casting, as shown in Fig. 1(a). Next, laser interference lithography was conducted to form PHPs on the PR layer, as depicted in Fig. 1(b) (see the experimental section). Subsequently, the UV, blue, and green LEDs with the patterned PR were etched through inductive coupled plasma. (Figure 1(c)) Finally, we obtained periodic holes on the LED surface after removing the PR, as shown in Fig. 1(d). Figure 1(e) presents the 3D schematic of the final LED structure with the PHPs. A fully fabricated single LED chip includes n-GaN as an electron injection layer, MQWs as an active layer, p-GaN as a hole injection layer, ITO as a transparent current-spreading layer, and Cr/Ar electrodes for both n- and p-GaN, as represented in the 3D schematic. Periodically arranged holes on the LED surface were confirmed by implementing FE-SEM. Figure 1(f) shows the plane-view SEM image of the patterned PR on the ITO surface, in which the PR holes on the ITO surface reveal periodicity with a square lattice pattern. The size of the holes and the periodicity of the patterns are 300 and 450 nm, respectively. The hole depth is approximately 40 nm. (see Supplementary Information, Fig. S1) Fig. 1(g) presents the plane-view SEM image of the ITO edge region, which is the border between the ITO and p-GaN electrical safety margin area. The inset shows the magnified SEM image of the p-GaN surface. The SEM images confirm that periodic holes were formed on not only the ITO surface but also the exposed p- and n-GaN regions. Figure 1(h)-(i) depict the photographs of the UV, blue, and green LEDs under static forward current. The applied current for the UV LED is 20 mA, and the applied current for the blue and green LEDs is 5 mA. This indicates that the light output power, which is a typical characteristic of InGaN-based LEDs, of the UV LED is less than those of the blue and green LEDs.

**Figure 2:**
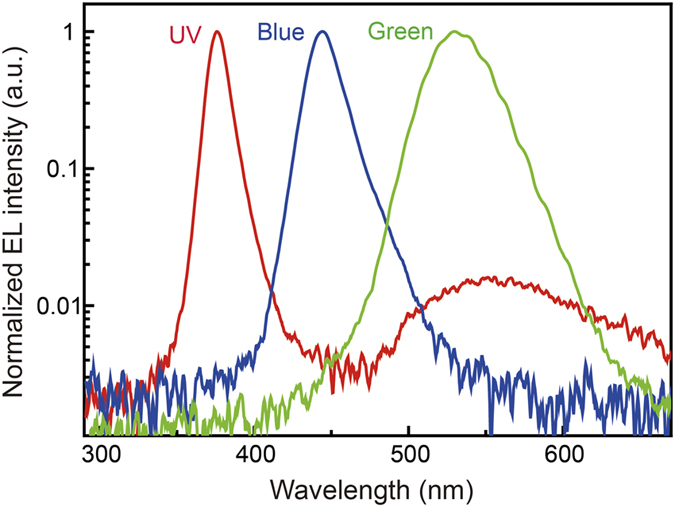
Emission properties of UV, blue, and green LEDs. Intensity-normalized EL spectra of the InGaN-based UV, blue, and green LEDs.

The emission properties of the InGaN-based UV, blue, and green LEDs were investigated using macro-electroluminescence (EL) spectroscopy. (see Supplementary Information, Fig. S2) Fig. 2 shows the intensity-normalized EL spectra of the UV, blue, and green LEDs used in this study. The peak wavelengths of the UV, blue, and green LEDs are 376, 445, 530 nm, respectively. The different full widths at half maximum of the EL peaks according to the emission wavelength are attributed to different In compositional fluctuations in the InGaN active layer. In EL spectrum of UV LED, the broad emission band revealed at wavelength range between 500 and 700 nm can be explained by defect-related yellow luminescence.^36^ This indicates that the emission properties of the conventional UV, blue, and green LEDs are typical and have representative wavelengths of UV, blue, and green lights.

**Figure 3:**
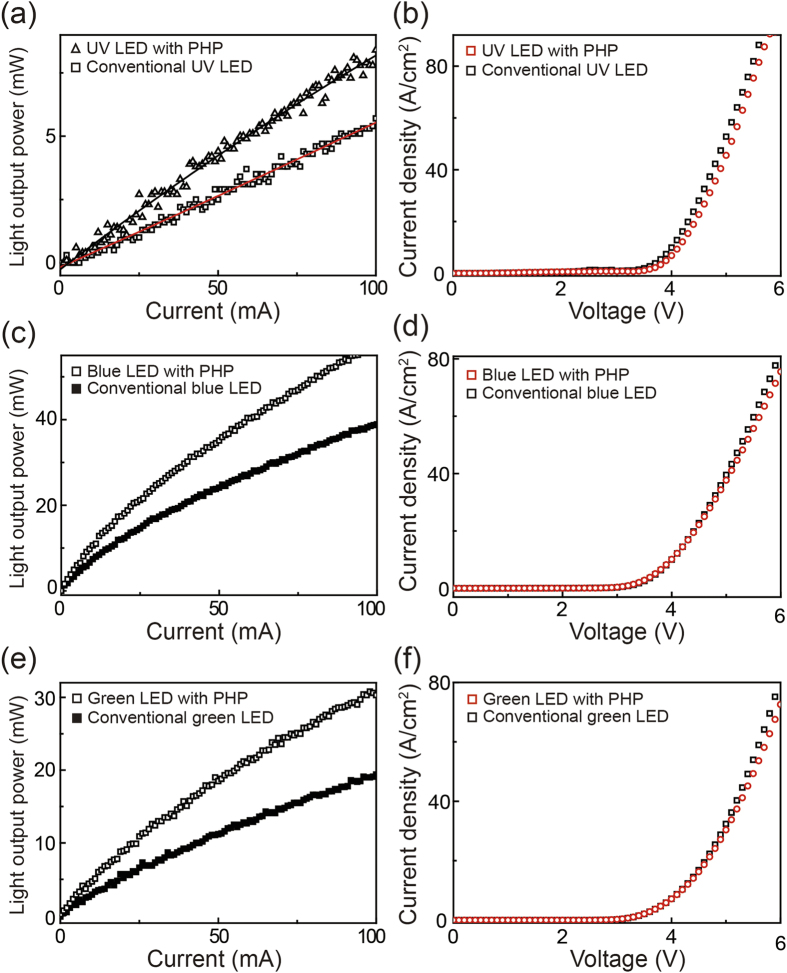
Device performance of the InGaN-based UV, blue, and green LEDs with and without PHPs. The L–I curves of (**a**) the UV, (**c**) blue, and (**e**) green LEDs with and without PHPs. Considerably different enhancements in the light output powers was observed according to the emission wavelength. The I–V curves of (**b**) the UV, (**d**) blue, and (**f**) green LEDs with and without PHPs. Implementation of PHPs shows slight changes in the electrical properties in all the LEDs.

The light output power versus the injection current (L–I) and current–voltage (I–V) curves of the UV, blue, and green InGaN LEDs with PHPs were measured to define the device performance. The injection current in the L–I curves ranges from 0 to 100 mA for the all samples. For the I–V curves, the applied voltage was swept from 0 to 10 V. Figure 3(a) shows the L–I curve of the UV LEDs with and without PHP. The hollowed triangle and square indicate the data points for the UV LEDs with and without PHP, respectively. Each data point was fitted through a linear equation because optical power fluctuations were considerably high. The black and red solid lines indicate the fitted L–I curves of the UV LEDs with and without a PHP, respectively. The light output power of the UV LED with PHP increased by 40% compared with that of the UV LED without PHP at an operating current of 20 mA. Figure 3(b) displays the I–V curves of the UV LEDs with and without PHPs, whose respective data points are indicated by the hollowed red and black squares. The series resistance and threshold voltage of the UV LED with PHP is slightly higher than those of the UV LED without PHPs; this can be explained by the plasma damage of the LED surface during dry etching. However, the I–V curves demonstrate slight changes in the electrical properties because the etching depth for a PHP is rather small (40 nm). (see Supplementary Information, Fig. S3) Fig. 3(c) and (e) demonstrate the L–I curves of the blue and green LEDs with and without PHPs. The hollowed and filled black squares indicate the data points for the LEDs with and without PHPs, respectively. The light output power of the blue LED with PHP increased by 50% at an operating current of 20 mA compared with that of the conventional blue LED. However, a 70% light output power increase is observed in the L–I curve of the green LED with PHP. Different absolute values of the light output power as a function of the emission wavelength are a typical feature of InGaN-based LEDs.^37^,^38^ Figure 3(d) and (f) respectively show the I–V curves of the blue and green LEDs, in which the hollowed red and black squares indicate the data points for the LEDs with and without PHPs, respectively. The figures show slight changes in the electrical properties of the blue and green LEDs with the application of PHPs. These slight changes in the I–V characteristics could be interpreted through a considerably small depth of holes on the LED surface. Based on experimental data, the calculated external quantum efficiencies (EQEs) of conventional UV, blue, and green LEDs at 100 mA are 5.5%, 38.9%, and 19.4%, respectively. Meanwhile, the EQEs of the UV, blue, and green LEDs with PHP are increased up to 8.2%, 58.3%, and 30.6%, respectively. Those EQE values are somewhat lower than that of recently reported maximum values.^9^,^39^,^40^ Internal quantum efficiencies of the UV (43.0%), blue (64.5%), and green (12.7%) LEDs, even though these are lower than reported maximum values^41^,^42^,^43^, are almost not changed due to significantly small hole depth of 40 nm. Therefore, we can conclude that the increases of EQEs are induced by enhanced LEE From the L–I–V measurements, we determined that the enhancement in the light output power of the LEDs with PHPs is sensitive to the emission wavelength and a small depth of holes significantly affects the light output power of the LEDs, with only limited slight changes in the electrical properties.

**Figure 4:**
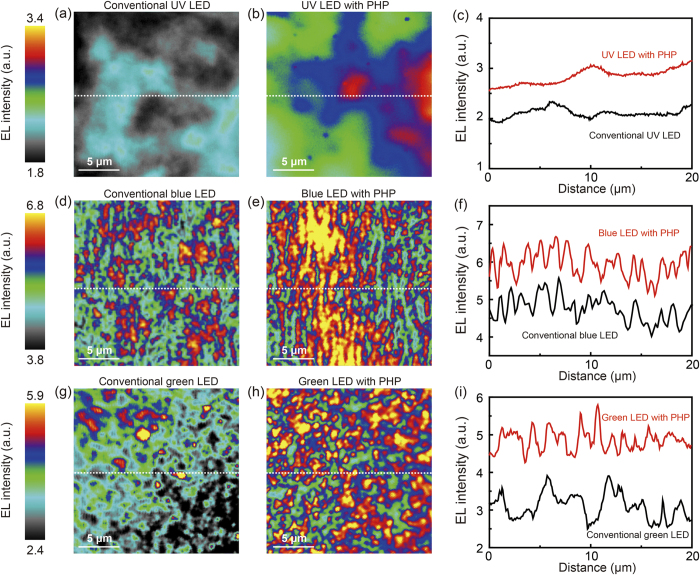
2D CSEM images and analyses of the UV, blue, green LEDs with and without PHPs. Static forward currents of 20, 1, and 1 mA were applied to the UV, blue, and green LEDs, respectively. The CSEM images of the conventional (**a**) UV, (**d**) blue, and (**g**) green LEDs without PHPs. The EL distributions are considerably different for the conventional UV, blue, and green LEDs. The CSEM images for the (**b**) UV, (**e**) blue, and (**h**) green LEDs with PHPs. The regions of the increased EL intensity are in accordance with the carrier-localized area. The EL intensity profiles of the (**c**) UV, (**f**) blue, and (**i**) green LEDs with and without PHPs.

To demonstrate the EL distribution of the UV, blue, and green LEDs with and without PHPs through a high spatial resolution, CSEM was employed.^44^ During CSEM scanning, accurately static currents of 20, 1, and 1 mA were applied to the UV, blue, and green LEDs, respectively. The focal plane of CSEM was fixed on the top surface, and the scan size was 20 × 20 μm for all the samples. The scale bar placed on the left side of the CSEM images indicates EL intensity. Figure 4(a) and (b) display the CSEM images of the UV LEDs without and with PHP, both of which show uneven EL intensity distributions attributed to indium compositional fluctuation in InGaN/GaN MQWs.^45^ However, the absolute EL intensity of the UV LED with PHP is higher than that of the conventional UV LED. The EL intensity profiles along the dotted white lines marked in Fig. 4(a) and (b) are plotted in Fig. 4(c). The red and black plots indicate the UV LEDs with and without PHPs, respectively. On average, a higher EL intensity profile is observed for the UV LED with PHPs than for the UV LED without PHP. Figure 4(d) and (e) demonstrate the CSEM images of the blue LEDs without and with PHP, respectively.

The localized bright spots, which have a size of approximately a few hundred nanometers, are randomly distributed over the entire scanning area for both CSEM images. This is attributed to carrier localization originating from the localized indium rich InGaN area, which typically occurs in blue and green InGaN/GaN MQWs.^46^ The CSEM image of the blue LED with PHP exhibits a higher EL intensity and larger area of bright spots than that of the conventional blue LED. The plots of the EL intensity profiles along the white dotted lines marked in Fig. 4(d) and (e) are displayed in Fig. 4(f). The EL intensities of the blue LEDs with and without a PHP are plotted using red and black lines. The plots reveal a considerably higher EL intensity fluctuation along the cross-sectional line for the blue LEDs compared with that for the UV LEDs, which corresponds to carrier localization induced by indium rich InGaNQWs.^47^,^48^ Moreover, the average EL intensity of the blue LED with PHP is noticeably higher than that of the conventional blue LED, as shown in Fig. 4(f). Figure 4(g) and (h) present the CSEM images of the green LEDs without and with PHPs, respectively. Inhomogeneous and localized bright spots which are larger than those of the blue LEDs were discovered in both CSEM images. This can be attributed to carrier localization caused higher indium contents in green MQWs.^48^,^49^ The EL intensity profiles marked along the white dotted lines in Fig. 4(g) and (h) are shown in Fig. 4(i). The red and black solid lines indicate the EL intensities of the green LEDs with and without PHP. As shown in Fig. 4(i), more peaks reflecting specific bright spots were observed for the green LED with PHP than for the conventional green LED. This indicates that more photons localized in indium-rich InGaN QWs were extracted to the air through the PHPs. The CSEM results show that the enhancement in the LEE is remarkably influenced by the emission wavelength and the indium contents of InGaN/GaN MQWs.

**Figure 5:**
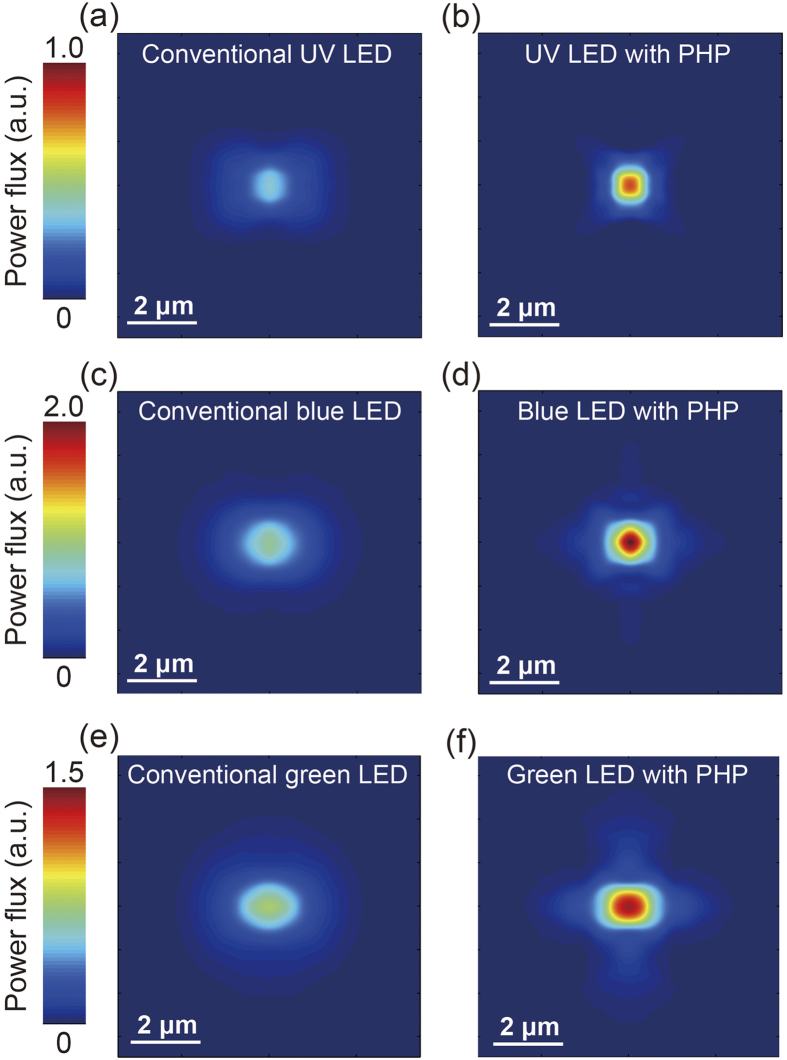
FDTD simulation images of the optical power flux for the conventional (**a**) UV, (**c**) blue and, (**e**) green LEDs without PHPs. The absolute optical power of the blue LED is higher than that of the UV and green LEDs. The FDTD images for the (**b**) UV, (**d**) blue, and (**f**) green LEDs with PHPs. The enhancements in the optical power fluxes of the UV, blue, and green LEDs with PHPs are considerably different.

The light output of the UV, blue, and green LEDs with and without PHP were simulated to confirm the experimental results. The 3D FDTD method was used to calculate the optical power emitted by the LEDs.^50^,^51^ For the boundary condition, we employed perfectly matched layers. An arbitrary mesh was used with 3 nm-sized grids near the edge of the materials along the *x*-, *y*-, and *z*-axes. To calculate the optical power flux (i.e., the *y*-component of the Poynting vector) through the surface of the LED, a plane top monitor was placed at (*x, y*_top_, *z*). Four monitors were placed above the device surface at the four side faces to obtain the total optical power. The optical power emitted by a single dipole source oriented in the three orthogonal directions was separately simulated because an LED is a randomly polarized light source. Finally, we summed all the monitored optical power. Figure 5(a–f) depict the FDTD images for the UV, blue, and green LEDs with and without PHPs. The colour-scale bars placed on the left side of the FDTD images indicate the optical power flux monitored from the UV, blue, and green LEDs. The optical powers revealed in the colour-scale bars for the UV, blue, and green LEDs are considerably different; this is consistent with the experimental results of the L–I curves and CSEM. Figure 5(a) and (b) respectively display the FDTD images calculated for the optical power flux of the UV LEDs without and with PHPs. The calculated optical power of the UV LED with PHP is distinctly higher than that of the conventional UV LED. This corresponds to the experimental data of the L–I curves represented in Fig. 3(a). Figure 5(c) and (d) show the FDTD images for the blue LEDs without and with the PHP, respectively. The absolute value of the optical power of the blue LEDs is higher than that of the UV and green LEDs, as presented in the colour-scale bars. Moreover, a significantly increased optical power flux was monitored from the blue LED with PHP than from the conventional blue LED. The enhancement in the optical power of the blue LED with PHP is higher than that in the optical power of the UV LED with PHPs; this is consistent with the experimental results of the L–I curves. Figure 5(e) and (f) respectively illustrate the FDTD images for the green LEDs without and with a PHP. The FDTD image of the green LED with PHP displays a drastically increased optical power flux than that of the conventional green LED; this corresponds to the experimental results, as previously shown in Figs 3 and 4. Furthermore, the augmentation of the optical power flux calculated for the green LEDs is considerably higher than that of the UV and blue LEDs. This is also consistent with the experimental results. Consequently, all the FDTD results for the UV, blue, and green LEDs with and without PHPs confirm the experimental data of the L–I curves and CSEM. The theoretical results calculated using the FDTD method confirmed that the enhancements in the light output power of the InGaN LEDs are significantly sensitive to the emission wavelength.

**Figure 6:**
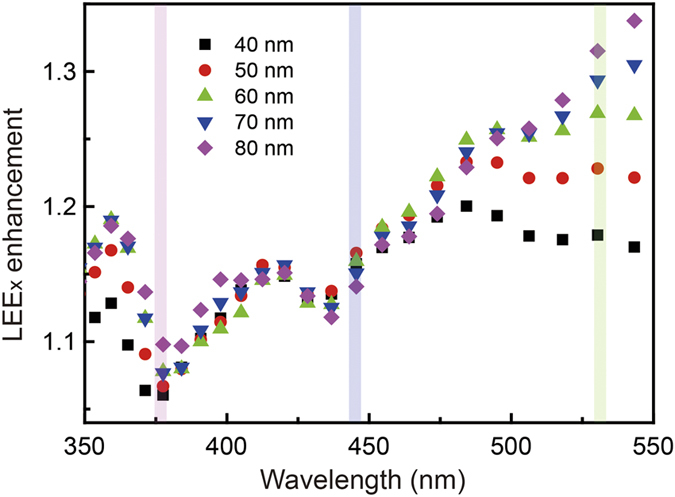
LEE enhancement factors of the InGaN-based LEDs with PHPs as a function of the emission wavelength. At wavelengths longer than 500 nm, the LEE enhancement is significantly influenced by the hole pattern depths on the LED surface.

To investigate the influence of the hole depth on the LEE of the InGaN LEDs with PHPs, we calculated the LEE enhancement factors according to the emission wavelengths and depths of the hole patterns. Figure 6 presents the LEE enhancement factors as a function of the emission wavelength calculated using the FDTD method. The black square, red circle, green triangle, blue reverse triangle, and purple lozenge indicate the data points for depths of 40, 50, 60, 70, and 80 nm of the hole patterns, respectively. We did not calculate the LEE for a hole depth larger than 90 nm to exclude the photonic crystal effect on the LED surface. The emission wavelengths of the UV, blue, and green LEDs studied in this research are marked using blurred purple, blue, and green lines respectively, in Fig. 6. According to the calculated data, the LEE enhancement factor is considerably influenced by the emission wavelength of the LEDs. Moreover, the tendency of the LEE enhancement factor for a depth of 40 nm, which is used in this study, according to the emission wavelength is in agreement with the experimental results of the L–I measurements. As shown in Fig. 6, the LEE enhancement factors of the UV and blue LEDs are not considerably influenced by the depth of the hole patterns. However, the influence increases with the emission wavelength.

Consequently, the LEE enhancement of the green LED with PHP is severely influenced by the depth of the hole patterns, as presented in Fig. 6. Since effective refractive index is defined according to the wavelength of the light, number of waveguide modes can be changed by emission wavelength of the LED. Moreover, the number of waveguide modes is sensitive to the thickness of the medium. For these reasons, the LEE enhancement of the green LED is more responsive to the depth of the hole pattern. This indicates that the light output power of the green LED can be simply modified by using a PHP, although a photonic band gap is not formed. The theoretical results of the LEE enhancement factors according to the emission wavelength and hole pattern depths confirm the experimental data and present an efficient method to improve the light output power of InGaN LEDs using PHPs. While having a perforated thin film on the top of device, one could also evoke a better light extraction through a refractive index matching layer. Indeed, as shown by the Fig. S4 in the supplementary information, a top layer of an intermediate effective refractive index allows to account qualitatively for the wavelength nevertheless a pure effective refractive index approach cannot reproduce the LEE enhancement observed experimentally. This clearly confirms that while interference effects have to be taken into account, the major effect here in terms of light extraction is due to light scattering via PHPs. In addition, we observed enhanced EL intensities detected from the p- and n-GaN layers of the UV, blue, and green LEDs owing to the formation of PHPs, indicating that the final enhancement in the light output power is partially contributed by this enhancement. (see Supplementary Information, Fig. S5).

## Discussion

In this study, we experimentally and theoretically investigated the enhancements in the light output powers of InGaN-based UV, blue, and green LEDs with PHPs. The emission wavelengths of the UV, blue, and green LEDs are respectively 376, 445, and 530 nm, which were verified through macro-EL spectroscopy. The PHPs were fabricated through laser interference lithography and ICP etching on the top surface of the LEDs. Periodic holes on the LED surface were examined using plane-view SEM images. The L–I and I–V curves show the different enhancements in the light output powers of the UV, blue, and green LEDs with PHPs, with slight changes in the electrical properties. The influence of the emission wavelength and indium contents of InGaN/GaN MQWs on the LEE enhancement was verified through the CSEM results. The FDTD simulation confirmed the experimental results of the enhancement in the light output powers. The calculated plots for the LEE enhancement factors according to the emission wavelength and hole pattern depths present optimal conditions for the PHPs to enhance the light output power of the InGaN LEDs without the photonic crystal effect. We believe that our experimental and theoretical study of the light output power of InGaN LEDs with PHPs will pave the way to realize low-cost high-efficiency full colour lighting.

## Methods

### Fabrication of InGaN-based LEDs

By using metal–organic vapour deposition, epitaxial structures of InGaN-based UV, blue, and green LEDs were grown on *c*-plane sapphire substrates. Trimethylgallium, trimethylindium, and ammonia were respectively used as precursors for Ga, In, and N in a reactor. To realize the emission wavelengths of UV, blue, and green light, the flow rate of trimethylindium in the growth process of MQWswas modulated. An indium tin oxide (ITO) transparent conducting layer and Cr/Au electrodes for both n- and p-type GaN layers were fabricated on the LED surface to complete an LED single chip.

### Formation of periodic hole patterns

To fabricate PHPs on the surface of the LEDs, laser interference lithography was conducted. A positive photoresist (PR) was spin-coated onto the LED surface. Further, these LED samples were baked at 110 °C for 1 min. As an illumination source, a He–Cd laser (λ = 325 nm) was applied. Half of the laser beam irradiated the PR after being reflected by the Lloyd mirror and the other half was directly projected onto the PR. The interference induced by the two beams caused a sinusoidal exposure on the PR, which occurred again after rotating the sample by 90° to achieve a square lattice pattern. The PR exposed by laser interference was removed by dipping the sample into a developer. The samples with the patterned PR were etched using inductive coupled plasma. After removing the PR by using acetone, we obtained periodic holes on the UV, blue, and green LEDs.

### Characterization

To observe the morphology of the LEDs with PHPs, FE-SEM (S-4700, Hitachi) was performed. To study the electrical properties and light output power of the LEDs with and without PHPs, a conventional probe station equipped with a Si photodiode and power supplier (SourceMeter 2400, Keithley) was used. The spectroscopic verification of the UV, blue, and green LEDs was performed using an EL spectrometer, consisting of a 30 cm monochrometer (SP2300, Princeton Instruments) and a thermoelectrically cooled charge-coupled device (PIXIS 100, Princeton Instruments). For spatially resolved EL analysis with a high spatial resolution, a confocal scanning EL microscope, consisting of an optical microscope, lead zirconate titanate nanopositioner, optical fiber, and optical power meter, were employed.

## Additional Information

**How to cite this article:** Jeong, H. *et al*. Indium gallium nitride-based ultraviolet, blue, and green light-emitting diodes functionalized with shallow periodic hole patterns. *Sci. Rep.* **7**, 45726; doi: 10.1038/srep45726 (2017).

**Publisher's note:** Springer Nature remains neutral with regard to jurisdictional claims in published maps and institutional affiliations.

## Supplementary Material

Supplementary Information
