# Supplementary material for: Indium gallium nitride-based ultraviolet, blue, and green light-emitting diodes functionalized with shallow periodic hole patterns
Source: Sci Rep. 2017 Apr 4;7:45726. doi: 10.1038/srep45726 (PMC5379558; doi:10.1038/srep45726)
Supplement: Supplementary Information [file srep45726-s1.doc]

Supplementary Information

Indium gallium nitride-based ultraviolet, blue, and green light-emitting diodes functionalized with shallow periodic hole patterns

Hyun Jeong^1,3^, Rafael Salas-Montiel^1^, Gilles Lerondel^1,2^, Mun Seok Jeong^2,3,*^

*Correspondence to [mjeong@skku.edu]

^1^Laboratoire de Nanotechnologie et d’Instrumentation Optique, Institut Charles Delaunay, CNRS-UMR 6281, Université de Technologie de Troyes, BP 2060, 10010 Troyes, France, ^2^Department of Energy Science, Sungkyunkwan University, Suwon 440-746, Republic of Korea, ^3^Center for Integrated Nanostructure Physics (CINAP), Institute for Basic Science (IBS), Sungkyunkwan University, Suwon 440-746, Republic of Korea.

**S1. Atomic force microscopy analysis for periodic holes formed on Indium tin oxide**

To estimate the surface morphology of periodic holes in the indium tin oxide (ITO) layer, the topography has been assessed using atomic force microscopy (AFM). Fig. S1(a) presents the topographical image of the ITO surface after fabricating the periodic hole patterns (PHPs); periodic holes are observed. Fig. S1(b) shows the height profile along the white dotted line marked in the AFM topographical image. The periodicity and depth of the PHPs are 450 and 40 nm, respectively. This confirms that the PHP formed in the ITO surface is periodic and the hole depths are sufficiently small to avoid the photonic crystal effect.


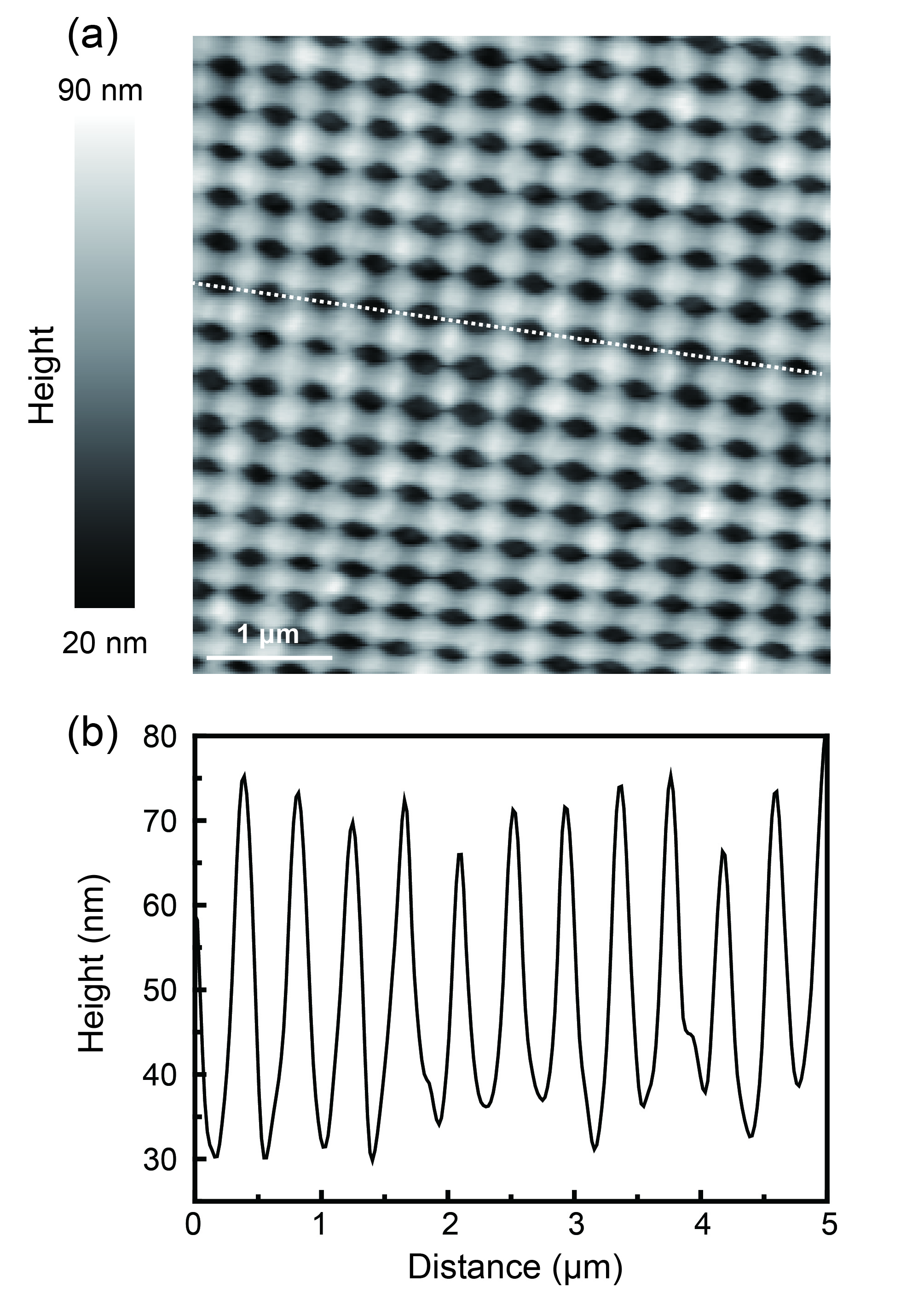


**Figure S1.** AFM topography of the PHP on ITO. (a) Topography of the ITO surface. (b) Height profile according to the white dotted line marked in the topography. The periodicity of the patterns and the hole depths are 450 and 40 nm, respectively.

**S2. Optical power and electroluminescence (EL) measurement systems**

Fig. S2 shows simplified schematic of optical power measurement (left) and EL spectroscopy (right) systems. For the measurement of optical power of the LEDs, we have used conventional probe station equipped with a power supplier (Keithley, SourceMeter 2400) and calibrated Si photodiode as presented in Fig. S2. The objective lens (NA = 0.5) placed above the sample collects the light emission of the LEDs and it delivers the light to the photodiode to detect the optical power. For EL spectroscopy, a 30 cm monochromator was applied to disperse the light collected from the sample. The thermoelectrically cooled charge-coupled device equipped with monochromator was used to detect optical signal as depicted in Fig. S2.


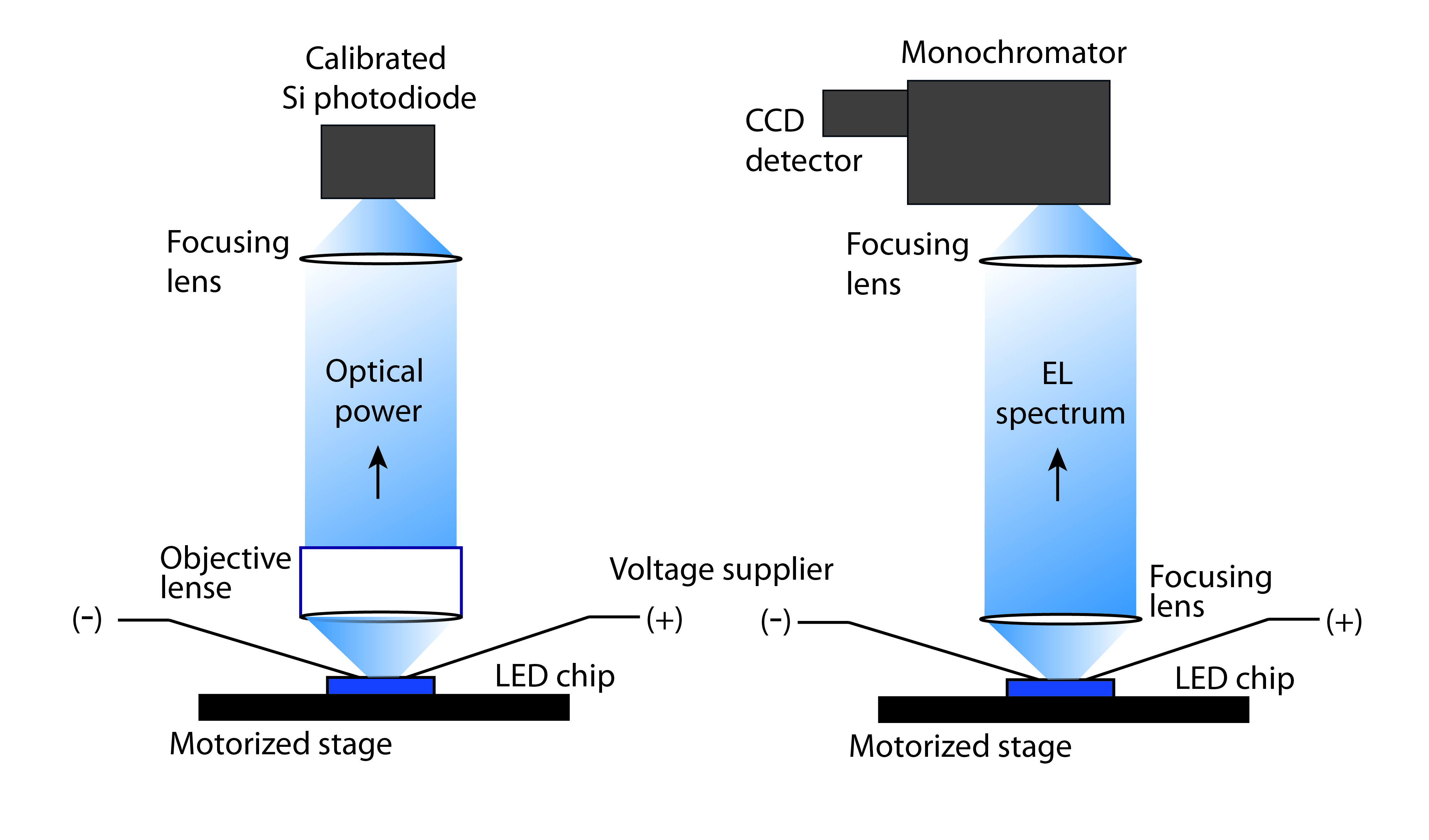


**Figure S2.** Simplified schematics of optical power and EL spectrum measurement systems.

**S3. Current-voltage (I-V) curves of the LEDs with and without PHP**

The I-V curves of the UV, blue, and green LEDs were plotted in log scale for observation of leakage current in the reverse bias. For all the I-V curves, the red circle and black square indicate I-V curves of LEDs with and without PHP, respectively. The voltage ranges is from -10 to 10 V. Fig. S3(a), (b), and (c) show I-V curves of UV, blue, and green LEDs, respectively. The leakage currents observed in UV, blue, and green LEDs were not considerably different by means of PHP. These results support that the electrical properties of LEDs were not noticeably affected by formation of PHP on the LED surface.


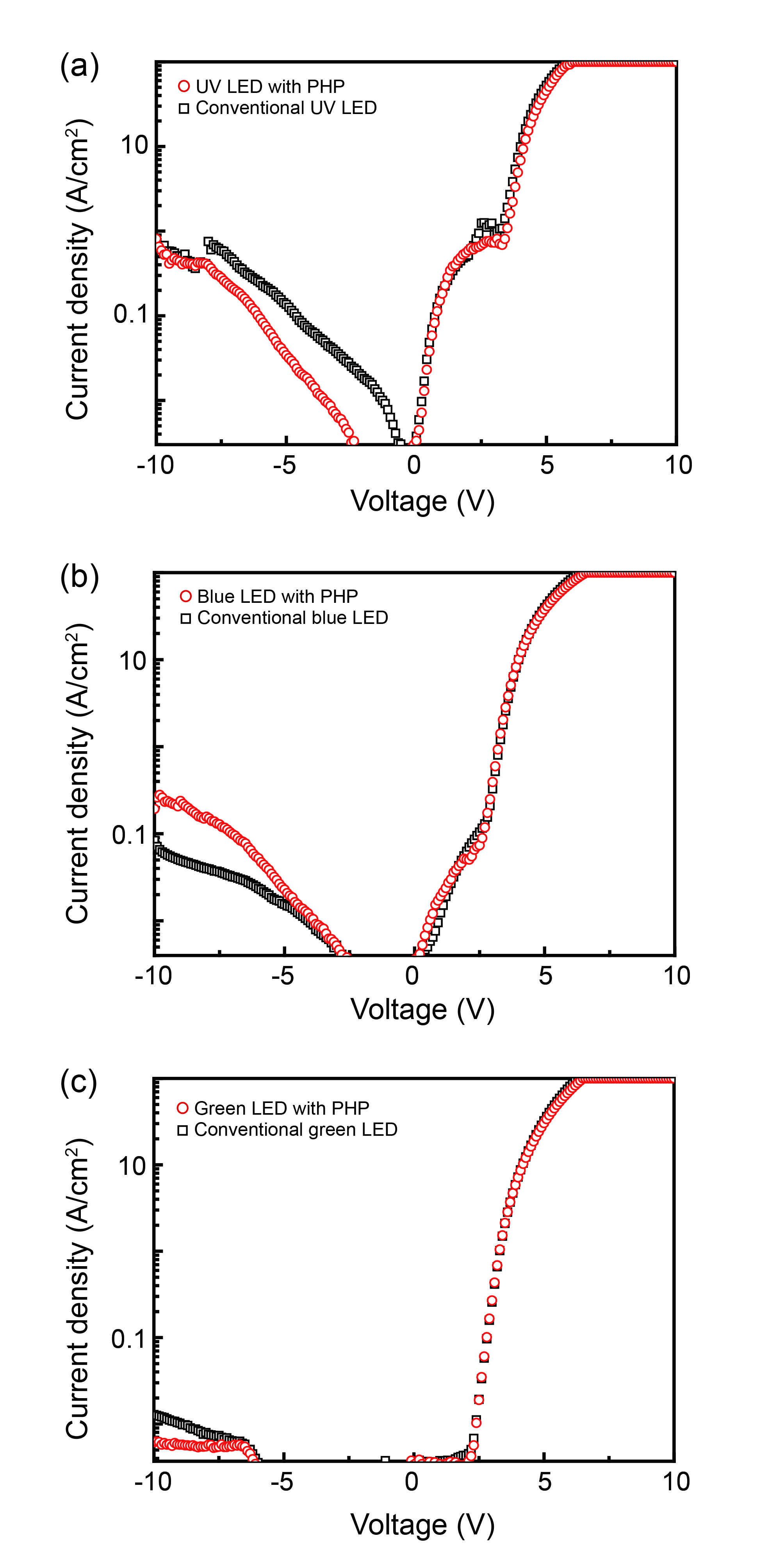


**Figure S3.** I-V curves of (a) UV, (b) blue, and (c) green LEDs with and without PHP plotted by log scale.

**S4. LEE enhancement of PHP LED calculated using effective medium theory**

The LEE enhancement factors of PHP LEDs were calculated using the effective medium theory to support the FDTD simulation results. The PHP area was regarded a slab consisting of air (holes) and ITO. The effective refractive indices of a PHP slab are calculated based on Maxwell Garnett, effective medium approximation (EMA), and Sellmeier models. Fig. S4(a) shows the LEE enhancement factors as a function of emission wavelength. The data points conforming to each hole depth are presented with different colours and shapes. Fig. S4(b) shows the LEE enhancement factors calculated by the EMA model. Fig. S4(c) exhibits the LEE enhancement plot simulated by Sellmeier model. Although the absolute values of LEE enhancement factors of an LED with PHP are slightly different according to each model, the trend of plots seems similar. For the blue LED, the LEE enhancement factor is not considerably influenced by the hole depth. However, LEE enhancements of the UV and the green LEDs are significantly influenced by the hole depth, as revealed in Figs. S4(a)–S4(c). This trend is also analogous to Fig. 6 in the main text. This implies that LEE enhancement of an LED with PHP can be explained through interference effects at least qualitatively. As far as absolute values are concerned, light scattering needs to be involved to explain the higher absolute values observed on the hole structured LEDs. This calculation clearly confirms that the LEE enhancement can not only be explained by a refractive index matching effect.


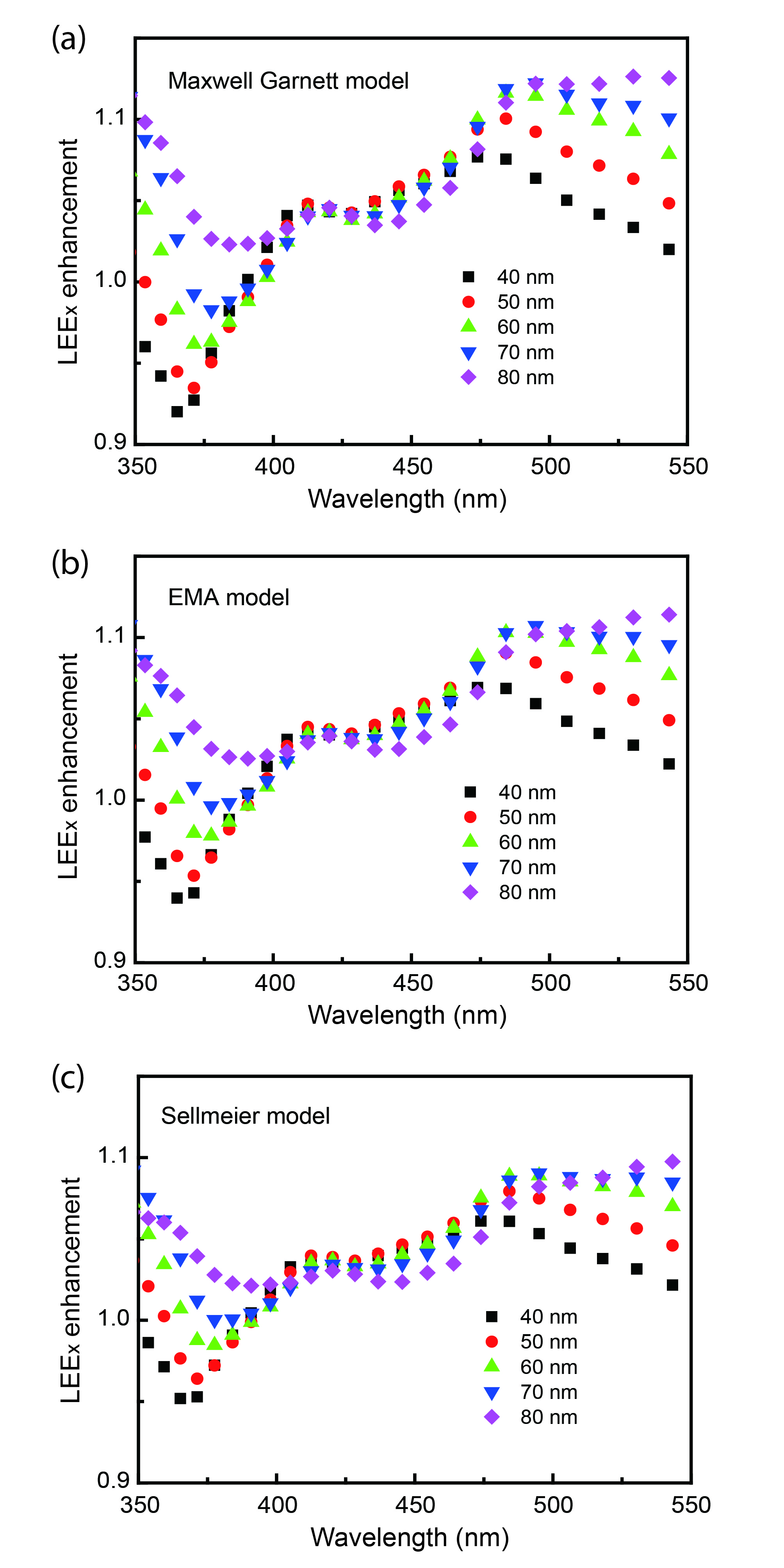


**Figure S4.** LEE enhancement plots for LEDs with PHPs as a function of the emission wavelength simulated using the effective medium theory. The LEE enhancement factors were calculated based on (a) Maxwell Garnett model, (b) EMA model, and (c) Sellmeier model.

**S5. Confocal scanning electroluminescence microscopy images of the edge region of the LEDs**

To confirm the influence of the PHPs formed on the p- and n-GaN layers of the LED structures, we employed confocal scanning EL microscopy (CSEM). Figs. S5(a) and S5(b) are the CSEM images of the UV LEDs without and with the PHP respectively, with the p- and n-GaN regions marked. The EL intensity increased at not only ITO but also the p- and n-GaN layers. The CSEM images of the blue LEDs without and with the PHP are demonstrated in Figs. S5(c) and S5(d), respectively. Similar to EL intensities in the UV LED, the EL intensities of the p- and n-GaN layers of the blue LED increased because of the PHP. Figs. S5(e) and S5(f) show the CSEM images of the green LEDs without and with the PHP, respectively. The EL intensities detected from the p- and n-GaN layers of the green LED were enhanced by the formation of PHPs, indicating that this enhancement partially contributed to the final enhancement in the light output power.


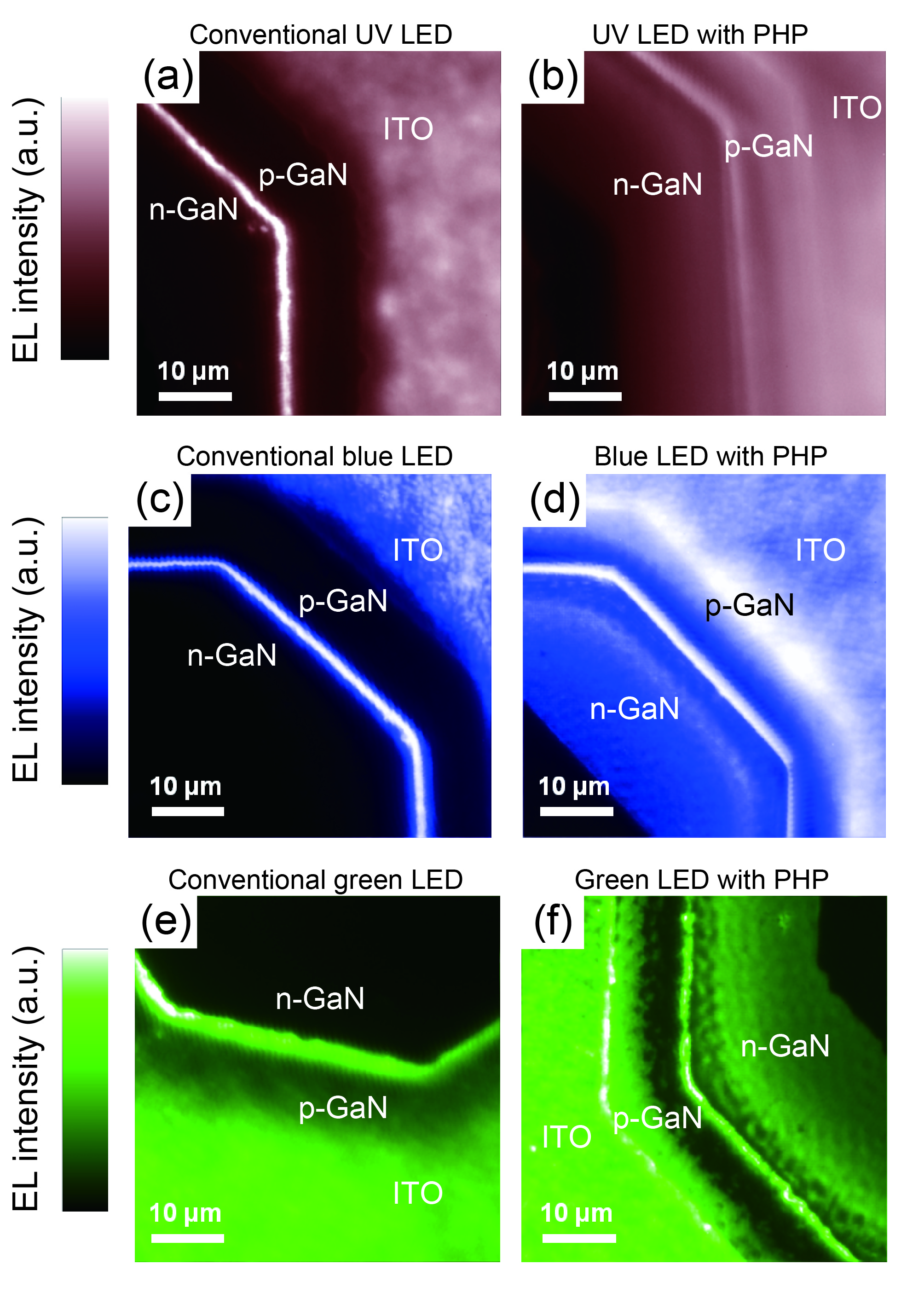


**Fig. S5:** CSEM images of the edge regions of the UV, blue, and green LEDs. The CSEM images of the UV LEDs (a) without and (b) with the PHP. The CSEM images of the blue LEDs (c) without and (d) with the PHP. The CSEM images of the green LEDs (e) without and (f) with the PHP. The enhancement in light extraction occurs at not only ITO but also the p- and n-GaN layers.
